# ^125^I seed implantation for lymph node metastasis from radioactive iodine-refractory differentiated thyroid carcinoma: a study on short-term efficacy and dosimetry

**DOI:** 10.3389/fonc.2024.1325987

**Published:** 2024-06-26

**Authors:** Wenwen Zhang, Shanhu Hao, Zhiguo Wang, Tingting Ding, Guoxu Zhang

**Affiliations:** ^1^ College of Medicine and Biological Information Engineering, Northeastern University, Shenyang, Liaoning, China; ^2^ Department of Nuclear Medicine, General Hospital of the Northern Theater Command, Shenyang, China

**Keywords:** thyroid tumor, tumor metastasis, lymph nodes, brachytherapy, iodine radioisotope, radiation dose

## Abstract

**Objective:**

To investigate the feasibility and evaluate the safety and effectiveness of Computed Tomography (CT) guided^125^I radioactive particle implantation for treating lymph node metastases in radioiodine-refractory differentiated thyroid cancer (RAIR-DTC). To verify the accuracy of the computerized three-dimensional treatment planning system (TPS) in treating lymph node metastasis using^125^I particle implantation at the dosimetric level.

**Methods:**

A retrospective analysis was conducted on 42 patients with RAIR-DTC and lymph node metastases who were admitted to the General Hospital of the Northern Theater Command between December 2016 and January 2019. During this analysis, physicians utilized preoperative CT images to design an intraoperative plan using TPS. The dosimetric parameters of the postoperative plan were then compared to the preoperative plan. Additionally, this study examined the changes in tumor size and tumor-related marker Thyroglobulin (Tg) values in patients at 2, 6, and 12 months after the operation.

**Results:**

The number of^125^I radioactive particles implanted in 42 patients was 226, with an average of 14.5 (range 2.0–30.0) particles implanted per lesion. The local remission rates were 97.62% (41/42), 88.10% (37/42), and 85.71% (36/42) at 2, 6, and 12 months postoperatively, respectively. The volume of the lesions was (4.44 ± 1.57) cm^3^, (4.20 ± 1.70) cm^3^, and (4.23 ± 1.77) cm^3^at 2, 6, and 12 months after treatment, respectively, which significantly decreased from the preoperative baseline level of (6.87 ± 1.67) cm^3^(*t*-values: 9.466, 9.923, 7.566, all *P*<0.05). The Tg levels were 15.95 (5.45, 73.93) μg/L, 8.90 (2.20, 39.21) μg/L, and 6.00 (1.93, 14.18) μg/L at 2, 6, and 12 months after treatment, respectively, which were significantly lower than the preoperative baseline levels of 53.50 (20.94, 222.92) μg/L (*Z* values: -5.258, -5.009, -4.987, all *P* < 0.001). Postoperatively, Delivered to 90% of the GTV(D_90_) was slightly lower than the prescribed dose in 95.23% (40/42) of patients, but the difference was not statistically significant [(12,378.8 ± 3,182.0), (12,497.8 ± 1,686.4) cGy; *t*=0.251, *P*>0.05], and postoperative dose parameters delivered to 100% of the gross tumor volume (GTV)(D_100_) (6,881.5 ± 1,381.8) cGy, the volume percentages of GTV receiving 150% of the prescribed dose(V_150)_ (58.5 ± 18.40)%) were lower than the preoperative plan D_100_ (8,085.8 ± 2,330.0) cGy, V_150_ (66.5 ± 17.70)%; *t*-value=8.913 and 3.032, both *P*<0.05; the remaining indicators were not significantly different from the preoperative plan (the differences in the number of implanted particles, Planning Target Volume(PTV), the volume percentages of GTV receiving 100% of the prescribed dose(V_100_), Homogeneity Index(HI)were not statistically significant (*t/Z* = -0.593, -1.604, 1.493, -0.663, all *P*>0.05).

**Conclusion:**

Referring to the TPS preoperative plan, the^125^I particle implantation therapy for RAIR-DTC lymph node metastasis can achieve the expected dose distribution, ensuring precise short-term local tumor control efficacy.

## Introduction

Thyroid cancer is a common malignancy of the endocrine system, with differentiated thyroid carcinoma (DTC) accounting for about 90% of cases (1). Most DTCs have a positive prognosis after surgery, supplemented with postoperative thyroid stimulating hormone (TSH) suppression or radioactive iodine (RAI) therapy. However, some tumor cells lose their ability to absorb iodine or have reduced TSH receptor expression, resulting in RAIR-DTC, with a 10-year survival rate of less than 10% (2). Finding effective treatments for RAIR-DTC is a major focus in the field of thyroid cancer. Currently, commonly used methods to treat RAIR-DTC include targeted drug therapy, local re-operation, chemotherapy, external irradiation therapy, immunotherapy, and gene therapy (3). In some cases, lymph node metastases are difficult to detect or are located near vital anatomical structures (such as the main trachea or large blood vessels), making surgery impossible. In recent years, radioactive particle implantation has become a widely used method for locally treating malignant tumors, such as Prostate Cancer (4);Lung Cancer (5); Breast Cancer (6). The distribution of the radiation dose is the most crucial factor in determining the effectiveness of particle implantation (7). By utilizing a TPS, a reasonable preoperative implantation plan can be developed to achieve the desired dose distribution. In this study, we investigated the short-term effectiveness and adverse effects of ^125^I particle implantation for treating lymph node metastases in RAIR-DTC. We compared the preoperative and postoperative dosimetric results of computerized 3D TPS-assisted particle implantation to evaluate the accuracy of this technique in guiding the dose distribution for particle implantation treatment.

## Materials and methods

### Study patients

**Figure 1:**
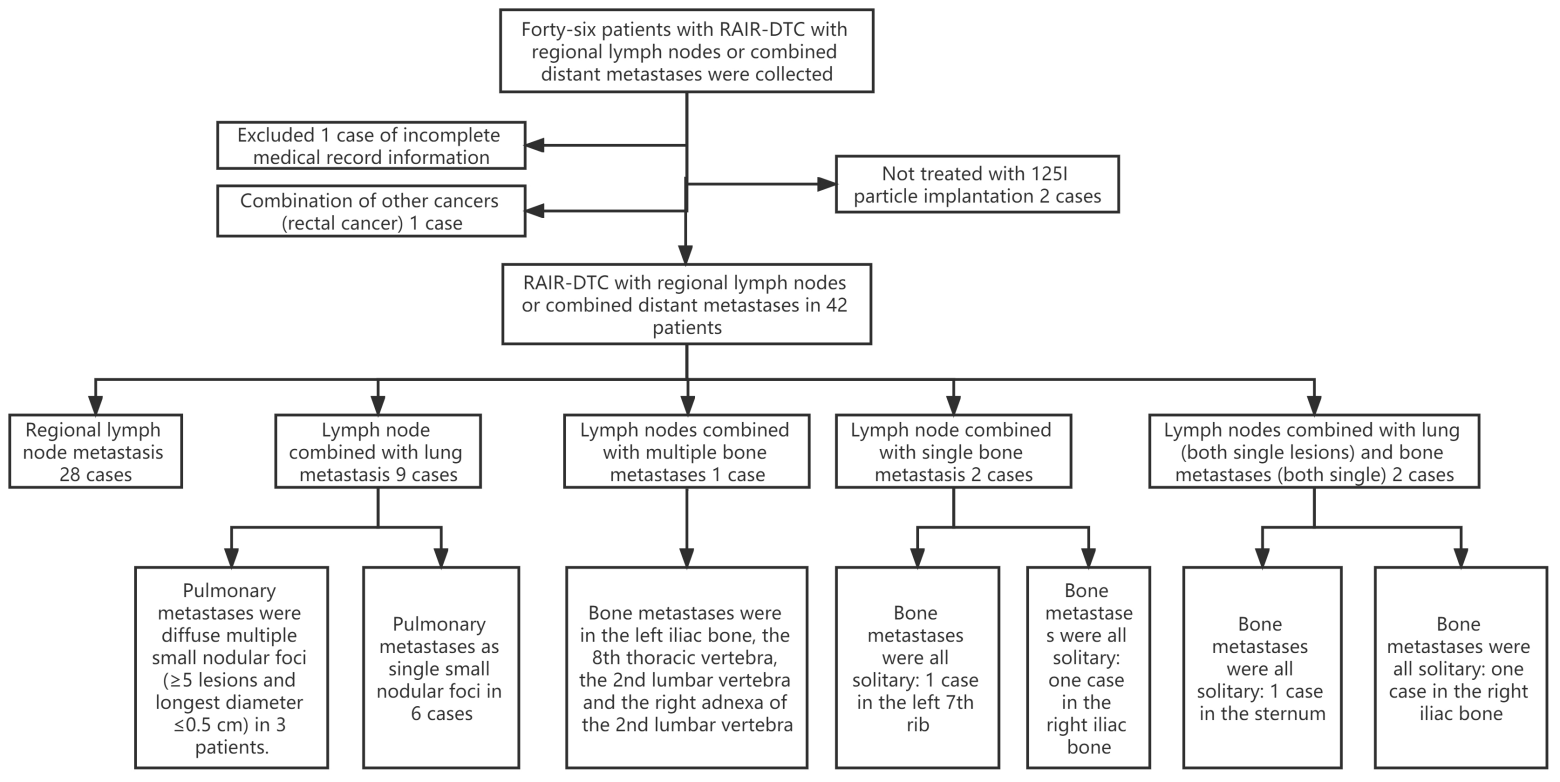
Flowchart of collecting patient data.

Forty-two patients with RAIR-DTC lymph node metastases, who were admitted and treated with ^125^I particle implantation at the General Hospital of the Northern Theater Command between December 2016 and January 2021, were selected for the study. One patient with incomplete medical records, two patients without ^125^I particle implantation, and one patient with rectal cancer were excluded. Ultimately, a total of 42 patients (21 males and 21 females) were enrolled, with an average age of 50.82 ± 12.79 years. Among them, two cases had lymph nodes combined with single bone metastasis (left 7th rib and right iliac bone), two cases had lymph nodes combined with lung metastasis (both single lesions), and two cases had bone metastasis (sternum and right iliac bone). Each patient received a cumulative dose of ^131^I >22.2 GBq, and the ^125^I particle implantation was performed at least six months after the ^131^I treatment. The study was reviewed and approved by the Medical Ethics Committee of our hospital [No: YLS No. (2019) 69], and all patients provided informed consent. The patient data collection process is shown in **Figure 1**.

### RAIR-DTC: radioiodine-refractory differentiated thyroid cancer

Inclusion criteria: (1) All patients who underwent total or near-total thyroidectomy (including extensive removal of cervical lymph nodes) before surgery, received postoperative ^131^I treatment, underwent biopsy of abnormal lymph nodes, and had confirmed metastasis of DTC through pathology. Metastatic lesions were confirmed using at least one imaging modality [such as ^131^I whole-body imaging, CT, Magnetic Resonance Imaging (MRI)] without iodine uptake. These patients were identified as having RAIR-DTC according to the 2015 American Thyroid Association (ATA) guidelines (8). (2) Patients with a white blood cell count (WBC) ≥3.5×10^9^/L (normal range: 3.5 to 9.5×10^9^/L), hemoglobin (Hb) ≥90 g/L (normal range: 130 to 175 g/L), prothrombin activity >40% (normal range: 70%~100%), platelet count (PLT) >100×10^9^/L (normal range: 125~350×10^9^/L), normal electrocardiogram (ECG), and liver function tests showing serum alanine aminotransferase and serum aspartate aminotransferase values not exceeding 3 times the upper limit of normal. (3) Patients with a Karnofsky performance status (KPS) score ≥70, detailed clinicopathological data, follow-up data, and a survival period of more than 1 year.

Exclusion criteria: (1) Patients with positive thyroglobulin antibody (TgAb). (2) Patients with severe cardiopulmonary, hepatic, or renal insufficiency. (3) Patients with severe coagulation dysfunction. (4) Patients with very poor general condition or cachexia.(5) Patients undergoing radiation and chemotherapy.(6)Patients undergoing immunotherapy.

### Instruments

①A Neu Viz 128 CT scanner from Neusoft Medical Group in Shenyang, China, was used to inject 0.1L of non-ionic contrast agent iodophoresol (Yangtze River Pharmaceutical Group, China; H10970358) at a flow rate of 2.5×10–^3^L/s through the patient’s elbow vein. The scan was delayed for 2 minutes, and the images were transmitted to the AW4.5 workstation provided by GE in the USA. The images were then transferred to the AW4.5 workstation for processing. ②The Discovery VCT PET/CT from GE in the USA was configured as a 64-row CT scanner. ③The Radioactive Particle TPS version 2.3 from Guangzhou Air Vision in China was used. ④The ^125^I radioactive particle source was produced by Beijing Atomic High Tech Co., Ltd. in China. It has a particle length of 4.5 mm, diameter of 0.8 mm, fully enclosed titanium shell, particle activity of 18.5~29.6 MBq, half-life of 59.6 days, and an average tissue penetration distance of 17 mm. The whole-body ^131^I imaging was performed using the Symbia T16 single photon emission computed tomography (SPECT) instrument from Siemens in Germany. Serum Tg was measured using a LIAISON chemiluminescence instrument from Sorin in Italy.

### TPS plan

**Figure 2:**
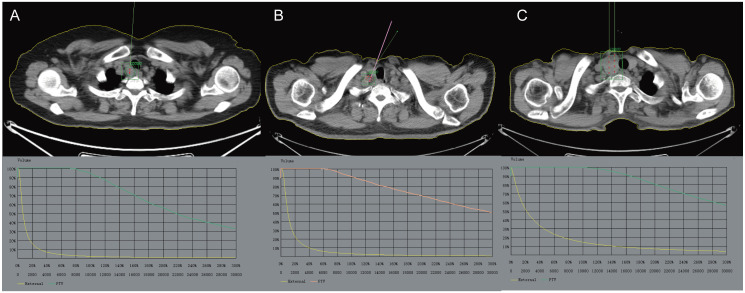
The preoperative CT scan images are imported into the TPS system for design, with the aim of avoiding important tissues and organs (such as large blood vessels, heart, spinal cord, etc.) as much as possible. **(A)** Enlarged right cervical lymph nodes with increased metabolism, indicating possible lymph node metastases. **(B)** Multiple lymph nodes in the right neck are enlarged and some of them have increased metabolism. **(C)** Occupation of the right lobe of the thyroid gland with increased metabolism is considered a local recurrence of thyroid cancer after surgery. The GTV and PTV are outlined. TPS, Treatment planning system; GTV, Gross Tumor Volume; PTV, Planning Target Volume.

The CT scan images taken one week prior to surgery are imported into the TPS system for design, with the aim of avoiding important tissues and organs (such as large blood vessels, heart, spinal cord, etc.) as much as possible. The GTV and the PTV are outlined, with the PTV range including the tumor itself and the surrounding tissues that may be infiltrated (e.g. peritumor burr sign). The appropriate prescription dose of 110–140 Gy and particle activity (14.8–25.9 MBq) were set (9), following the principles of particle spacing of 0.5–1.0 cm, needle tract spacing of 1 cm, and a “sparse in the center, dense around” approach. By optimizing the prescription dose D_90_ > matched peripheral dose (MPD), the optimal needle route was simulated by calculating the number of particles and particle distribution source. The preoperative planning parameters D_90_ and D_100_, the volume percentages of GTV receiving 100% and 150% of the prescribed dose (V_100_ and V_150_), and the dose-volume histogram (DVH) were obtained. As shown in **Figure 2**.

### The surgical procedure

Different positions were chosen based on the location of the lesion. An extracorporeal positioning grating was placed on the skin area corresponding to the lesion. The puncture site was determined using a CT scan. Local infiltration anesthesia was administered by injecting lidocaine with a mass fraction of 2%. The depth and angle of the puncture path were determined, and the needle was inserted layer by layer according to the TPS plan. Subsequently, one particle was implanted every 0.5–1.0 cm in a backward manner, replenishing the particles from the periphery to the center. The needle was then repositioned based on the position of the implanted needle. Care was taken to avoid important blood vessels and nerves during the implantation process. Throughout the procedure, the patient’s heart rate, blood pressure, respiration, and oxygen saturation were monitored. Additionally, the patient’s consciousness, pain, respiration, cough, and hemoptysis were observed, and timely symptomatic treatment was provided. After the surgery, the puncture site should be pressed for 10–20 minutes.

### Postoperative validation plan

Immediately after the surgery, the CT images were sent to the TPS for postoperative verification. This involved identifying particles and gathering statistics on D_90_, D_100_, V_100_, V_150_, DVH, and the HI. A higher HI indicates a more even distribution of the dose in the target area (10).

### Postoperative review and evaluation of recent efficacy

(1) Determination of efficacy: The efficacy of ^125^I particle implantation treatment was assessed by reviewing neck ultrasound and neck CT scans before and 6 months after treatment. The evaluation was based on the Response Evaluation Criteria in Solid Tumors (RECIST) 1.1 (11). (1) Complete remission (CR) was defined as the complete disappearance of lesions, with no imaging indicating recurrence, tumor remnants, or metastases, except for the presence of ^125^I particle metallic shadowing. (2) Partial remission (PR) was defined as a minimum 30% reduction in the sum of short diameters of target lesions compared to the sum of minimum diameters measured before treatment. (3) Stable disease (SD) was defined as a minimum 30% reduction in the sum of short diameters of target lesions compared to the sum of minimum diameters measured before treatment. (4) PD was defined as a ≥20% increase in the sum of the diameters of the target lesions compared to the minimum sum of the short diameters of the target lesions during the entire study, or an absolute increase of ≥5 mm in the sum of the diameters (the appearance of one or more new lesions was also considered as PD).

Adverse effects: Patients were closely monitored for adverse reactions such as bleeding, hematoma, particle displacement, and needle tract implantation after surgery. The Radiation Therapy Oncology Group/European Organization for Research and Treatment of Cancer (RTOG/EORTC) criteria were used to evaluate skin radiation injury caused by radioactive particle implantation, and complications during the intraoperative, postoperative, and follow-up periods were observed and assessed (12).

### Statistical analysis

SPSS 21.1 statistical software was utilized for data analysis. Quantitative data were assessed for normal distribution using the Kolmogorov-Smirnov test. Normal data were presented as (mean ± s), while paired samples t-test was employed for comparing two groups. Skewed data were expressed as median and range of values [M(P_25_, P_75_)]. The rank sum test was used for comparing two groups. Statistical data were presented as n(%). The χ^2^ test was utilized for comparing two groups. All tests were considered statistically significant at *P* < 0.05.

## Results

### Recent changes in efficacy and volume were observed after ^125^I particle implantation

**Figure 3:**
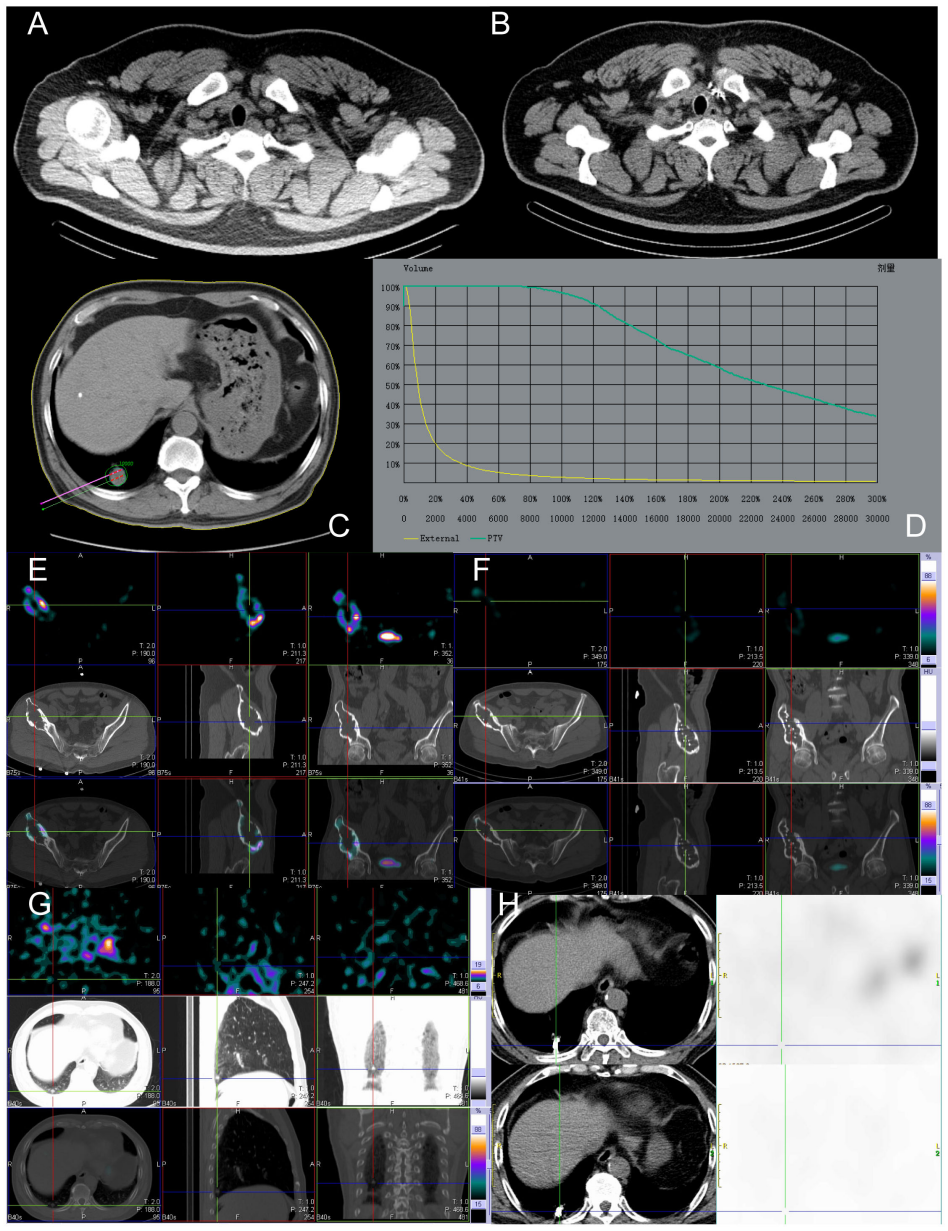
Before and after implantation of left supraclavicular lymph node, lung, and right ilium lesions with ^125^ I seeds. The patient, a 49-year-old male with RAIR-DTC, had postoperative pathologic findings of papillary thyroid carcinoma. **(A)** Images of ^125^ I seeds before implantation. **(B)** CT reexamination 12 months after the implantation of ^125^ I seeds showed only the shadow of metal seeds. **(C)** Preoperative needle placement of TPS system. **(D)** After the dose verification, the planned target DVH showed D_90_ > 100 Gy (prescription dose) and V_200_ < 60%, indicating satisfactory dose distribution of particle implantation with no “cold area” or “hot area” in dosimetry. **(E)** Single Photon Emission Computed Tomography/Computed Tomography (SPECT/CT) revealed iodine uptake in the right ilium metastatic lesion. **(F)** After 12 months of treatment, compared to before treatment, the radiation uptake significantly decreased, indicating effective local treatment, partial inhibition of tumor activity, and significant improvement in the patient’s bone pain. **(G)** No iodine uptake was found in the right lower lobe lung metastasis by SPECT/CT. **(H)** After 12 months of treatment, CT images showed a significant reduction in the mass and partial relief in the local effect. Tg, Thyroglobulin; RAIR-DTC:Radioiodine-refractory differentiated thyroid cancer; CT, Computed Tomography; TPS, Treatment planning system; DVH, Dose-volume histogram; SPECT/CT, Single Photon Emission Computed Tomography/Computed Tomography.

**Figure 4:**
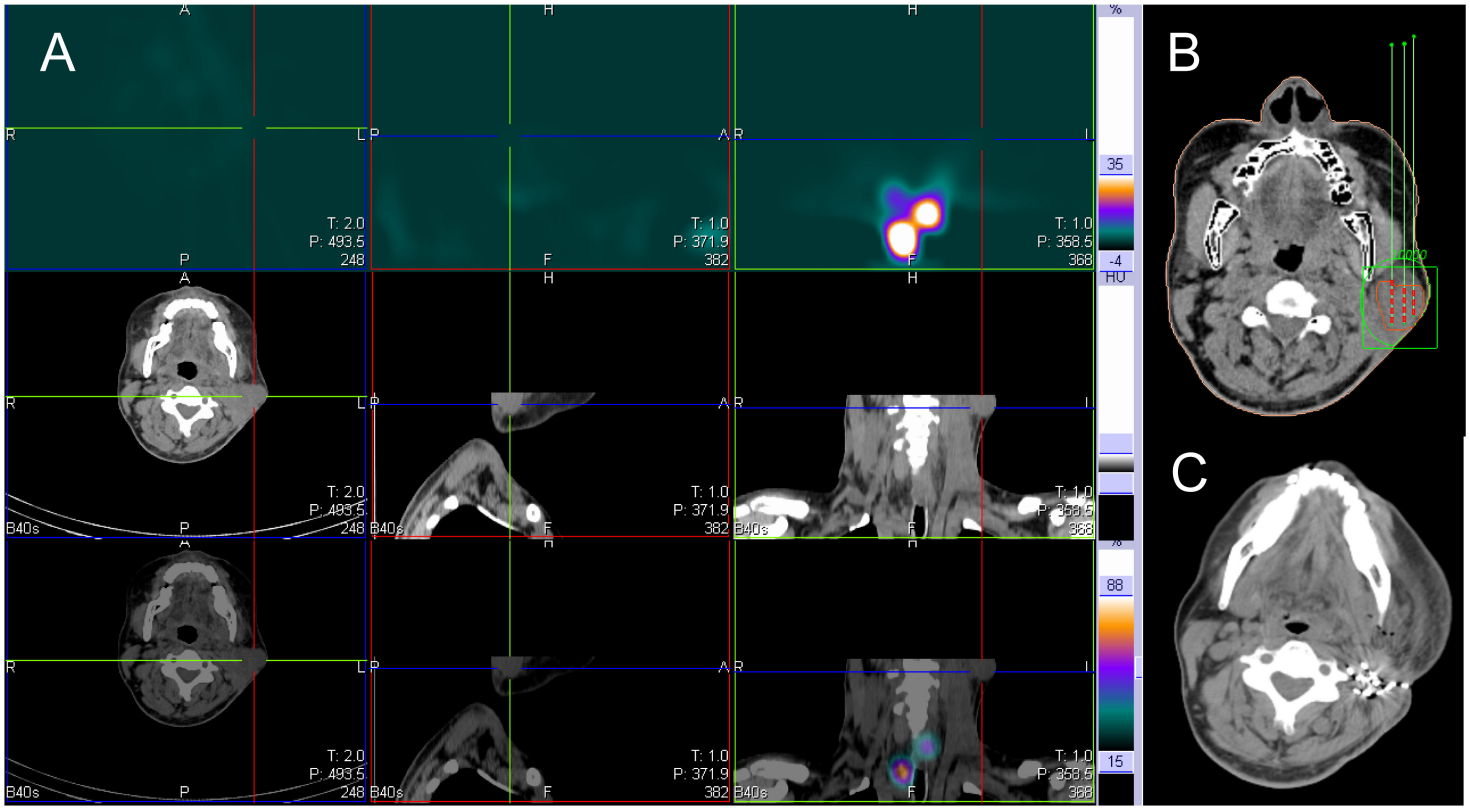
^131^I SPECT/CT imaging was performed on a 37-year-old male patient with ^131^I refractory differentiated thyroid cancer. The imaging revealed metastasis to the left neck lymph node, as indicated by the cross and arrow (shown in the image). **(A)** The ^131^I SPECT/CT neck fusion imaging showed no iodine uptake in the left cervical lymph node metastases. **(B)** The TPS was used for preoperative needle placement. **(C)** After 6 months, reexamination of the left cervical lymph node showed significant shrinkage, particle aggregation. SPECT/CT, Single Photon Emission Computed Tomography/Computed Tomography; TPS, Treatment planning system.

**Figure 5:**
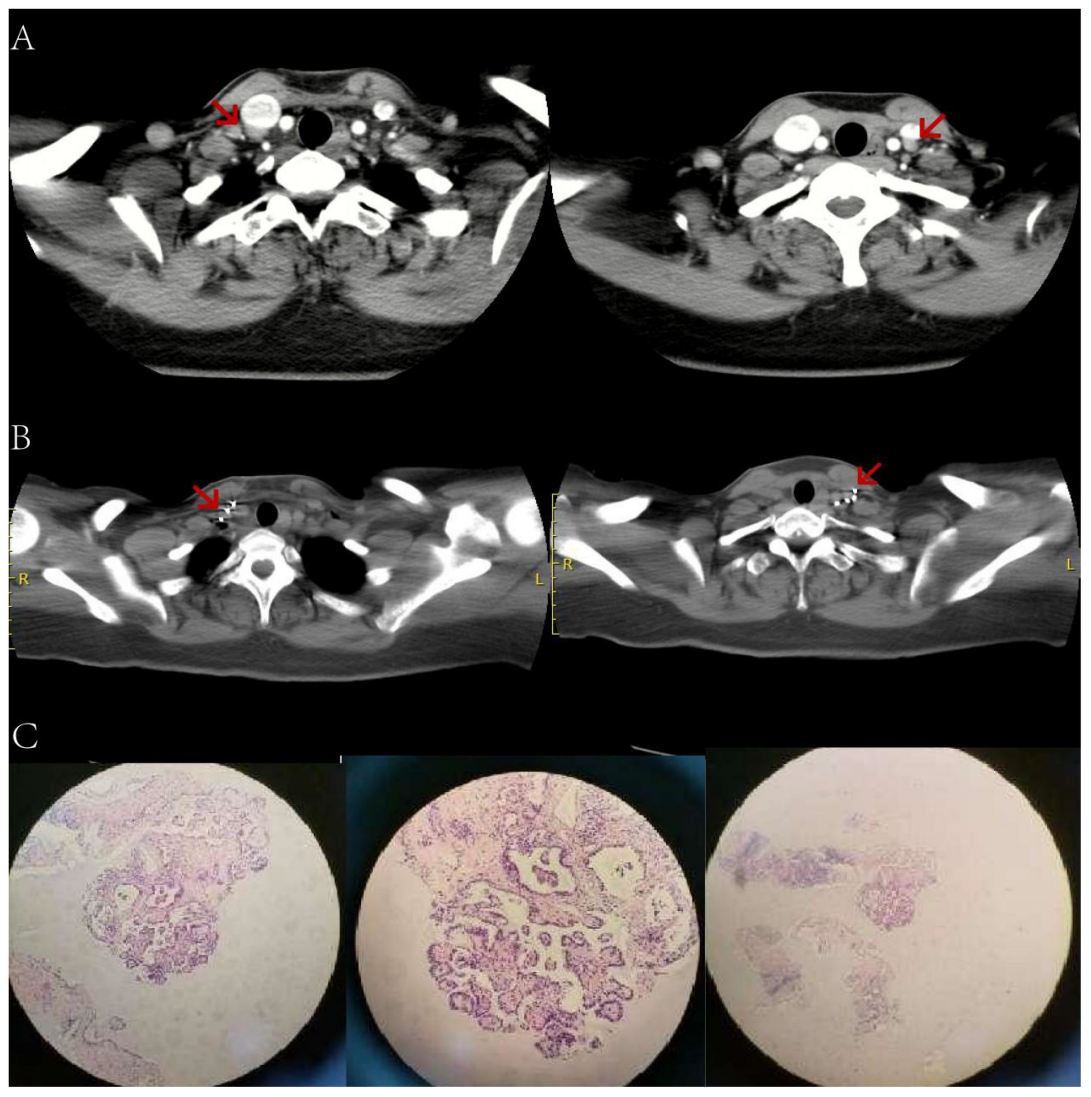
A female patient, aged 55, who underwent thyroid cancer surgery more than 2 years ago. **(A)** An enhanced CT scan of the neck reveals additional bilateral cervical lymph node metastases (indicated by arrows). The preoperative Tg level was 63.70ng/ml (in a TSH suppressed state). **(B)** 6 months after receiving 125 iodine radioactive particle implantation treatment, the lesion has significantly reduced in size compared to before. The Tg level is now 3.50ng/ml. **(C)** Pathology examination shows a small amount of epithelial cells in the tissue, indicating papillary severe heterogeneous hyperplasia. CT, Computed Tomography; Tg, Thyroglobulin; TSH, Thyroid stimulating hormone.

**Figure 6:**
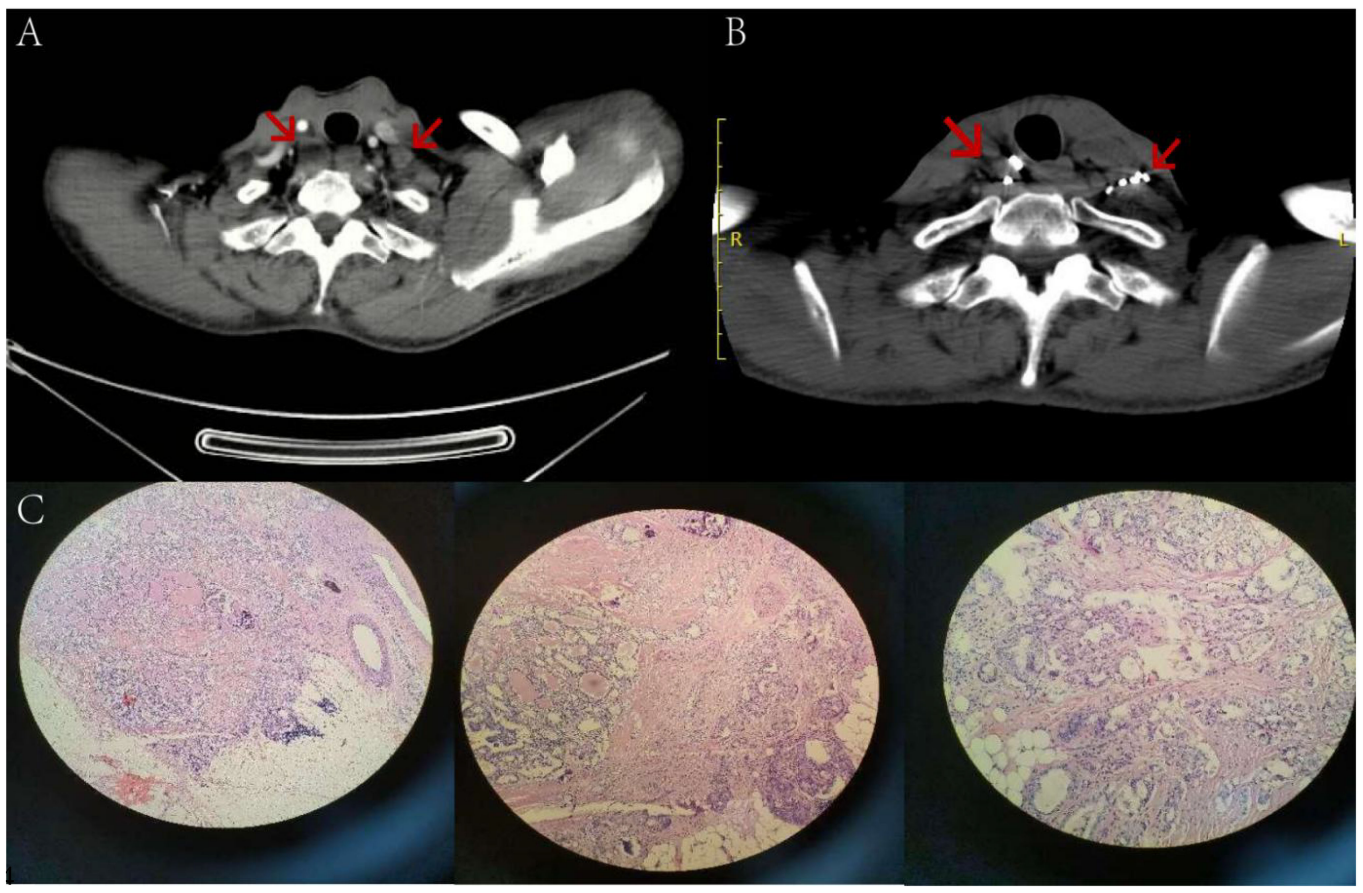
A 52-year-old male patient, who underwent thyroid cancer surgery over two years ago, has multiple lymph node metastases in both necks (indicated by arrows) as seen in image **(A)** Before the surgery, the patient had a preoperative Tg level of 56.50 ng/ml while in a TSH suppressed state. After 5 months of 125 iodine radioactive particle implantation treatment, the lesions have significantly reduced in size compared to before, as shown in image **(B)** The Tg level has also decreased to 8.21 ng/ml. Pathology examination reveals severe atypical hyperplasia of adenoid structures in the lymph nodes, which, when combined with immunohistochemical staining results, suggests metastatic adenocarcinoma of thyroid origin. Tg, Thyroglobulin; TSH, Thyroid stimulating hormone.

All 42 patients underwent successful implantation, with a range of single particle activity from 14.8 to 25.9 MBq, with an average of 14.5 (2.0~30.0) particles implanted per lesion. A total of 226 particles were implanted, and the number of particles implanted in all lesions was consistent with the distribution of the treatment plan, resulting in a compliance rate of 95.23% (40/42). Two lesions were not implanted as planned due to patient movement and the proximity of the tumor to large blood vessels in the neck. Follow-up evaluations were conducted at 2, 6, and 12 months after surgery to assess the efficacy of ^125^I particle implantation, as shown in **Table 1**. Further analysis revealed that the volume of the lesions significantly decreased at 2, 6, and 12 months after surgery compared to the pre-surgery volume of (6.87 ± 1.67) cm^3^ (*P*<0.05). Additionally, the Tg value was significantly lower at 2, 6, and 12 months after surgery compared to the pre-surgery value of [53.50(20.94, 222.92) μg/L] (*P*<0.05), as shown in **Table 2** (some patients had Tg values over 1000 μg/L or less than 0.2 μg/L). It is worth noting that the TgAb was negative in all cases. **Figures 3**–**6** display typical case images.

**Table 1:** Efficacy analysis of ^125^ I seed implantation at different time points before and after treatment.

Follow-up time	CR	PR	SD	PD	LCR%
2 months after surgery	4	35	2	1	97.62
6 months after surgery	6	27	4	5	88.10
12 months after surgery	6	26	4	6	85.71

CR, Complete remission; PR, Partial remission; SD, Stable disease; PD, progressive disease.

**Table 2:** Tumor volume and Tg value before and after treatment.

Items	Tumor volume(cm^3^ $\bar{\chi }$ ± s)			Tg value[μg/L, M(P_25_, P_75_)]		
	2 months after surgery	6 months after surgery	12 months after surgery	2 months after surgery	6 months after surgery	12 months after surgery
Measuring value	4.44 ± 1.57	4.20 ± 1.70	4.23 ± 1.57	15.95(5.45, 73.93)	8.90(2.20, 39.21)	6.00(1.93, 14.18)
*t/Z*	9.466	9.923	7.556	-5.258	-5.009	-4.987
*P*	<0.001	<0.001	<0.001	<0.001	<0.001	<0.001

Tg, Thyroglobulin.

### Analysis of factors influencing the recent efficacy

At 12 months post-surgery, 36 cases were categorized into the effective local control group (CR + PR + SD), while 6 cases were placed in the ineffective group (PD). This categorization was based on the evaluation criteria of RECIST _1.1_ combined with MDA bone metastases. The volume and degree of enhancement of the lesions prior to treatment, as well as the presence of distant metastases, were found to be associated with recent efficacy (all *P* < 0.05). However, factors such as gender, pathological type (papillary, follicular), age (≤45 years, >45 years), and preoperative Tg values did not significantly impact the outcome, as indicated in **Table 3**.

**Table 3:** Analysis of influencing factors of short- term treatment effect *n (%)/* $\bar{\chi }$ *± s*.

Items	Local control effective group	Local control invalid group	*χ^2^/t/Z*	*P*
Gender			0.778	0.378
Male	17 (40.5)	4 (9.5)		
Female	19 (45.2)	2 (4.7)		
Pathological pattern			0.032	0.857
Papillary thyroid carcinoma	31 (73.8)	5 (11.9)		
Follicular thyroid carcinoma	5 (11.9)	1 (2.4)		
Metastatic sites			7.875	0.005
Regional lymph node metastasis	27 (64.3)	1 (2.4)		
Merge distant transfer	9 (21.4)	5 (11.9)		
Age (years)			0.024	0.877
≤45	16 (38.1)	2 (4.8)		
>45	20 (47.6)	4 (9.5)		
Preoperative volume (cm^3^ )	3.55 ± 0.62	21.18 ± 6.82	-2.502	0.041
Preoperative enhancement (HU)	50.00 ± 3.45	21.33 ± 4.16	3.976	0.011
Preoperative Tg values (μg/L)	65.19 ± 37.30	202.8 ± 59.79	-1.007	0.332

### ^125^I particle implantation therapy achieves the desired dose distribution

Referring to the TPS schedule, the ^125^I particle implantation achieved the expected dose distribution (**Table 4**). Postoperatively, D_90_ was slightly lower than the prescribed dose in 95.23% (40/42) of patients, but the difference was not statistically significant (*t*=0.251, *P*>0.05). In terms of dosimetric indices, both D_100_ and V_150_ were lower than the preoperative plan (*t* value: 8.913, 3.032, both *P*<0.05); however, there were no statistically significant differences in the number of implanted particles, PTV, V100, and HI (*t/Z* =-0.593, -1.604, 1.493, -0.663, all *P*>0.05).

**Table 4:** Dose validation results were obtained before and after lesion particle implantation procedures in 42 patients diagnosed with iodine-refractory differentiated thyroid cancer.

Time	Pre-operative planning	Post-operative verification	*T/Z*	*P*
Number of implanted particles[grain;M (P_25_,P_75_)]	15.5 (2.0~32.0)	14.5 (2.0~30.0)	*Z* =-0.593	*P*=0.553>0.05
PTV[cm^3^; M (P_25_,P_75_)]	1.4 (0.6~9.8)	1.4 (0.6~10.6)	*Z* =-1.604	*P*=0.109>0.05
D_90_ (cGy;x ± s)	12497.8 ± 1 686.4	12378.8 ± 3 182.0	*t* =0.251	*P*=0.402>0.05
D_100_ (cGy;x ± s)	8085.8 ± 2 330.0	6881.5 ± 1 381.8	*t* =8.913	*P*=0.013<0.05
V_150_ (%;x ± s)	66.5 ± 17.70	58.5 ± 18.40	*t* =3.032	*P*=0.035<0.05
V_100_ (%;x ± s)	94.5 ± 8.27	92.2 ± 14.3	*t* =1.493	*P*=0.161>0.05
HI[M (P_25_,P_75_)]	0.3 (0.2~0.4)	0.4 (0.3~0.5)	*Z* =-0.663	*P*=0.508>0.05

D_90_ and D_100_ represent the prescribed doses received by 90% and 100% of the GTV, respectively. HI refers to the uniformity index, PTV stands for the planned target volume, and V_100_ and V_150_ indicate the percentages of the GTV that receive 100% and 150% of the prescribed dose, respectively. GTV: Gross Tumor Volume; PTV: Planning Target Volume; HI: Homogeneity Index.

Intraoperatively, there were no occurrences of serious bleeding or vascular embolism. One case experienced a small amount of intraoperative bleeding at the surgical site, which was successfully treated with intravenous injection of hemostatic drugs and compression to stop the bleeding. Additionally, two cases had grade II skin reactions, which improved after the administration of anti-inflammatory and topical drugs. Importantly, there was no migration of radioactive particles to other tissues or organs. Referring to the TPS preoperative plan, the ^125^I particle implantation therapy for RAIR-DTC lymph node metastasis can achieve the expected dose distribution with few complications, making it a safe and effective treatment method.

## Discussion

Cervical lymph node metastasis is the most common occurrence after Papillary Thyroid Carcinoma(PTC). The majority of these metastasis lesions are found near the large blood vessels in the neck, the trachea, and the area where important nerves run, and some tumor growth may invade surrounding tissues (13). The tumor focus grows rapidly and invades the surrounding tissues, leading to clinical symptoms such as facial edema, pain, hoarseness, and limb numbness. After multiple treatments with ^131^I, some patients develop RAIR-DTC. RDT may restore RAI avidity and induce RECIST response, particularly in those with RAS-driven ‘follicular’ phenotype, providing effective treatment for patients (14). Targeted drugs such as sorafenib and lenvatinib are also used as first-line treatments for RAIR-DTC, significantly prolonging progression-free survival but having minimal impact on overall survival. However, both treatment modalities exhibit significant drug resistance and adverse reactions, making them challenging to meet clinical needs (15). In recent years, the benefits of ^125^I seed therapy have been gradually explored in clinical settings. ^125^I continuously releases 27.4~31.4keV X-rays and 35.5keV γ-rays between tissues, with γ-rays directly causing breaks in the single-strand or double-strand DNA of the tumor, and X-rays inactivating tumor cells through the production of oxygen free radicals by indirect ionization (16).

Additionally, ^125^I particles act on some cancer cells in the quiescent phase (G_0_ phase), transforming them into proliferative phase (G_2_ and M phase) cells that are more susceptible to being killed after entering the mitotic phase (17, 18). Recently, ChenW (19) treated 15 patients with cervical recurrence after DTC using ^125^I seed therapy. The volume reduction rate was (83.5 ± 16.9)% 12 months after the procedure, and the postoperative serum Tg level was 4.9 (0.7,13.2) μg/L, significantly lower than the preoperative level of [57.0 (8.6,114.8) μg/L]. Furthermore, 22 high-enhancement foci transformed into low or no enhancement foci. In this study, among 17 patients who underwent ^125^I particle implantation therapy, there was 1 case of complete response (CR), 10 cases of partial response (PR), and 4 cases of stable disease (SD).The size of the lesions and the Tg levels after treatment were significantly lower than those before the procedure, consistent with the results of previous studies (19). After treatment, the symptoms of 3 cases of hoarseness and 5 cases of local tenderness were significantly relieved. In this study, fixed head support or human fixed body membrane was used to avoid and reduce patient displacement during implantation. The results of this study show that ^125^I radioactive seed therapy is minimally invasive, safe and can significantly improve the quality of life of patients in the short term. The results of this study show that ^125^I radioactive particle therapy is minimally invasive, safe, and can significantly improve the compression symptoms caused by cervical lymphadenopathy in the short term.

Dose determination is a crucial aspect of ^125^I seed implantation. The optimal dose distribution was automatically designed based on the tumor boundary, location of important tissues, prescription dose, and particle activity prior to TPS. After the procedure, the quality of radioactive particle implantation was assessed by identifying particles and verifying the discrepancy between the target dose and prescription dose. According to the Code for Clinical Application of radioactive particles in tumor treatment, the prescribed radiotherapy dose is set between 110 to 140Gy (20). If the dose is too low, it will fail to achieve the objective of tumor eradication; if the dose is too high, it will harm the normal tissues and organs surrounding the target area. In this study, there was no significant difference in dosimetric parameters such as D_90_, V_100_, and HI before and after the procedure. The postoperative D_90_ was (12378.8 ± 3182.0) cGy and V_100_ was (92.2 ± 14.3)%, indicating that the radioactive particle implantation essentially achieved the intended distribution and fulfilled the dosimetry requirements as per the preoperative TPS plan. However, in this study, the HI before and after the procedure is low, and the postoperative verification V_150_ exceeds 50%, suggesting an uneven dose distribution in the target area and an excessive volume of high-dose region. The possible causes of the errors were analyzed: (1) The change in focus size and the rapid reduction of perifocal edema accelerated the dispersion and regression of radiation dose, resulting in a decrease in the conformal index of dose distribution within the focus (21–27). Additionally, the postoperative verification dose does not reflect the actual absorbed dose of the tumor. In this study, the postoperative verification was conducted within 24 hours, and the local unsubsided tissue edema shortly after the operation affected the evaluation of the biological effect dose (25). (2) The accurate location identification of particles is challenging due to postoperative CT artifact, with one particle imaging at two levels and partial particle overlap, leading to dose deviation in postoperative verification measurements. (3) The density of the tumor varies due to bleeding or necrosis caused by the radiation effect of particles, resulting in changes in particle position. Furthermore, postoperative verification revealed disease progression in two patients approximately four months after implantation. Further investigation found that the postoperative D_90_ was significantly lower than the planned prescription dose before the operation. The main reasons for this are as follows: (1) The operation area structure in these two patients is disordered, their age is advanced, and the neck tissue is soft, which may have caused implantation deviation during the operation. (2) The shape of the target volume changed due to local muscle contraction caused by body position examination, intraoperative anesthesia, and puncture stimulation before and after the operation.

Most of the lesions in RAIR-DTC lymph node metastases are located near the body surface. The release of γ-rays by ^125^I seeds can lead to DNA double-strand breaks and impact the metabolism of skin cells. In this study, two patients experienced painful erythema and pigmentation, but no skin rupture occurred after one week of anti-inflammatory treatment. This was because many lesions were superficial and in close proximity to each other, resulting in the formation of local “hot spots” due to particle aggregation. It is recommended that when implanting particles, the distance between the particles and the skin’s surface layer should be at least 1cm greater, and the activity and number of particles should be adjusted based on the anatomical location of the lesions.

In summary, the treatment of RAIR-DTC lymph node metastasis using ^125^I seed implantation has the advantages of good efficacy and few complications. The use of TPS can improve the dose conformability of the target area, effectively killing the tumor, and better protecting the surrounding normal tissue. However, it is important to note that this study is a single-center retrospective study with a small sample size and a short follow-up cycle. Therefore, it is necessary to expand the sample size in order to analyze the effects of dose, tumor size, and other factors on the curative effect. This will provide better guidance for the standardized diagnosis and treatment of ^125^I seeds.

## Data availability statement

The raw data supporting the conclusions of this article will be made available by the authors, without undue reservation.

## Ethics statement

The study was reviewed and approved by the Medical Ethics Committee of our hospital [No: YLS No. (2019) 69]. The studies were conducted in accordance with the local legislation and institutional requirements. The participants provided their written informed consent to participate in this study. Written informed consent was obtained from the individual(s) for the publication of any potentially identifiable images or data included in this article.

## Author contributions

WZ: Writing – original draft, Funding acquisition. SH: Writing – original draft, Formal analysis, Methodology. ZW: Writing – original draft, Conceptualization, Project administration, Software, Validation. TD: Conceptualization, Project administration, Software, Validation, Writing – original draft, Data curation, Formal analysis, Investigation, Methodology, Resources, Supervision, Visualization. GZ: Resources, Supervision, Writing – original draft, Funding acquisition, Writing – review & editing.
